# Practical Step-by-step SYNAPSE VINCENT Rendering of Three-dimensional Graphics in Horseshoe Kidney with Bilateral Varicoceles

**DOI:** 10.31662/jmaj.2024-0058

**Published:** 2024-08-09

**Authors:** Kosuke Kojo, Jaejong Kim, Tsukasa Saida, Tomoyuki Ohta, Keisuke Sano, Shuya Kandori, Akio Hoshi, Hiromitsu Negoro, Hiroyuki Nishiyama

**Affiliations:** 1 Department of Urology, Institute of Medicine, University of Tsukuba, Tsukuba, Japan; 2 Center for IVF and Infertility, International University of Health and Welfare Hospital, Nasushiobara, Japan; 3 Tsukuba Clinical Research & Development Organization, University of Tsukuba, Tsukuba, Japan; 4 Department of Medical Education, Institute of Medicine, University of Tsukuba, Tsukuba, Japan; 5 Department of Radiology, Institute of Medicine, University of Tsukuba, Tsukuba, Japan; 6 Department of Radiology, International University of Health, and Welfare Hospital, Nasushiobara, Japan

**Keywords:** SYNAPSE VINCENT, SYNAPSE 3D, horseshoe kidney, bilateral varicoceles

## Abstract

Medical illustration serves as a cornerstone for understanding intricate anatomical anomalies, with three-dimensional (3D) rendering emerging as a pioneering tool for emphasizing basic medical concepts and clinical practices. In Japan, the SYNAPSE VINCENT software package (SVSP; Fujifilm Medical Co., Ltd., Tokyo, Japan), internationally known as “SYNAPSE 3D,” is a widely embraced solution for 3D rendering. However, despite its prevalence, resources elucidating its practical usage and offering insightful tips are scarce. In this review, we focus on the use of SVSP for 3D rendering of complex anatomical anomalies, particularly in the field of urology. We demonstrate a step-by-step process of 3D rendering. 3D rendering was performed in a sample case of a patient with horseshoe kidney and coexisting bilateral varicoceles through inputting of multiphase contrast-enhanced CT images into the application, followed by segmentation of the renal parenchyma, image registration, and segmentation of the arterial and venous systems as well as the upper urinary tract. Manual adjustments were made using the “Mask edit” and “Diameter setting” tools to ensure accuracy, particularly in cases of significant anomalies. Then, color-coded structures appeared, including the renal parenchyma, arterial and venous systems, and upper urinary tract, which provided a comprehensive visualization of the anatomical anomalies. This review highlights the effectiveness of the SVSP in visualizing complex anatomical abnormalities and detailing the practical rendering process, which could promote wider adoption of the application among urologists despite the challenges associated with the software.

## Introduction

Medical illustrations are important for understanding complex anatomical anomalies, with three-dimensional (3D) rendering constituting an innovative, illustrative tool for obtaining insights into basic medical considerations and clinical practice. The SYNAPSE VINCENT software package (SVSP; Fujifilm Medical Co., Ltd., Tokyo, Japan), marketed outside of Japan as “SYNAPSE 3D,” ^(1), (2), (3), (4), (5)^ is widely used in Japan. However, despite its widespread adoption for 3D rendering in Japan, practical resources and reviews on SVSP usage and tips are limited, other than the official manual, which is 8-cm thick ^(6)^.

Since 2018, our team has been using SVSP, mainly in urology, to generate high-quality 3D renderings of various organs, facilitating effective medical communication. By understanding the advantages and limitations of the excellent semiautomatic segmentation of SVSP, we observed that even individuals who lack artistic skills can generate visually appealing displays through straightforward operations without requiring deep understanding of complex graphical concepts.

We herein illustrate a practical, step-by-step rendering process using a sample case of a patient with horseshoe kidneys and bilateral varicoceles. Furthermore, we enrich our collection of tips with a narrative review of the technical literature.

## Sample Case Whose CT Images Were Used for 3D Rendering

### Initial assessment of the sample case

The sample case was a 45-year-old man who consulted the authors for infertility. Semen analysis of a 2.2-mL sample revealed a sperm concentration of 98 million/mL and a sperm motility of 2%, which led to the diagnosis of severe asthenozoospermia. A thorough physical examination revealed bilateral varicoceles, with the left varicocele being larger than the right one and visible even when the patient was in the recumbent position, indicative of dilated veins. The right varicocele was palpable only with the patient standing and performing Valsalva maneuver. Abdominal ultrasound performed to investigate the etiology of the varicoceles revealed the presence of horseshoe kidneys. However, the renal morphology and surrounding vasculature could not be fully visualized with ultrasound alone because of the presence of acoustic shadows caused by intestinal gas. Moreover, the patient reported unexplained chronic lower abdominal pain that persisted for years, particularly when experiencing increased abdominal pressure, such as during ejaculation. Furthermore, there were concerns that the varicoceles could be due to an underlying retroperitoneal tumor, including renal cancer. Considering the aforementioned factors and recognizing that ultrasound alone as a diagnostic modality might overlook the presence of such a tumor, additional imaging tests were conducted.

### Indications and imaging condition for CT examination

Because of concerns regarding the adverse effects of X-ray exposure on reproductive function, CT scans are excluded from our routine screening protocols for male infertility. However, in this case, after consultation with urologists regarding the aforementioned indications, it was found that the benefits of a CT scan outweighed the risks. After thoroughly explaining this to the patient and his family, a CT examination was conducted.

The CT scan was performed using an Aquilion ONE scanner (Canon Medical Systems Corp., Tochigi, Japan) with the following acquisition parameters: tube voltage of 120 kVp, X-ray tube current of 270 mA, detector collimation of 320 × 0.5, and slice thickness of 1 mm. Subsequently, Omnipaque 300 (GE Healthcare, Milwaukee, WI, USA), a dose of 600 mg of iodine per kg of body weight, was injected via the peripheral vein of the upper extremity over a 30-s period.

The arterial phase was initiated using the bolus tracking method and triggered when the region of interest (ROI) in the lumen of the abdominal aorta exceeded 150 Hounsfield units. Immediately after the arterial phase, approximately 40 s after initiating the contrast administration, the corticomedullary phase was reached. This was succeeded by the nephrographic phase at 90 s and the excretory phase, which was achieved 7 min after the injection.

### CT examination findings and detailed explanation provided to the patient

The CT scan confirmed the diagnosis of a horseshoe kidney with bilateral varicoceles but did not show any retroperitoneal tumors. Moreover, it excluded any other potential causes of lower abdominal pain besides the varicoceles and addressed the possibility of Kartagener syndrome, which could have been associated with the severe asthenozoospermia and presented symptoms such as situs inversus or bronchiectasis ^(7)^.

The CT findings, including the vascular structures around the horseshoe kidney, were visualized through 3D rendering, which enabled efficient information exchange between the urologists and the patient, who had various concerns. Thereafter, the patient and his wife underwent infertility treatment, which is not described herein. Informed consent was obtained from the patients, and this report was approved by the Institutional Review Board of the University Tsukuba Hospital (approval number: R03-033).

## SVSP Stepwise 3D Rendering

### Step 1: Input of multiphase contrast-enhanced CT images

We used SVSP version 6.70007 (server type), hosted on a server with the following specifications: a Lenovo ×3,500 M5 model, equipped with dual Intel Xeon Processor E5-2620 v3 (2.40 GHz, 15 MB cache, 1,866 MHz, 85W), 128 GB of RAM (16 × 8 GB/2R/2133 MHz/TruDDR4 RDIMM/CPU × 2), and 16 TB of storage (RAID6). The server runs on Microsoft Windows Server 2012 R2 Standard Edition (×64/10 Cal) and connects through an Intel I350-T4 1Gb Ethernet network. SVSP was accessed through a dedicated computer terminal installed in our hospital (Microsoft Windows 10 Professional, 64-bit, Intel Core i5-7500 CPU @ 3.40 GHz, RAM 16.0 GB DDR4) to perform 3D rendering. Of the greater than 50 versatile features of SVSP ^(8)^, we preferentially used the “Kidney Analysis” application, which semiautomatically reconstructs 3D graphics of the renal parenchyma and perirenal vascular structures ^(4), (5)^. We applied this feature in rendering.

**Figure 1.**
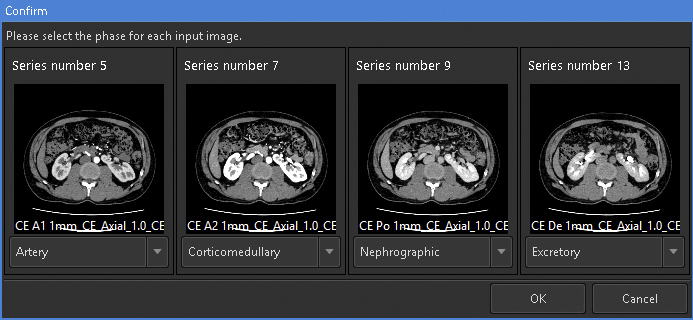
Initial screen of the “Kidney Analysis” application. When launching the application, we ensured that the image series to be analyzed matched the phases specified by the application. We prepared the arterial, corticomedullary, nephrographic, and excretory phases with 1-mm slice thickness and input them into the application.

For 3D rendering using this feature, the selection of appropriate images is crucial. This feature can simultaneously analyze up to four phases of same-session contrast-enhanced CT images. To render visually appealing images with minimal effort, it is recommended to prepare at least corticomedullary- and excretory phase images. To consistently obtain high-quality images, it is necessary to ensure minimal motion or respiratory artifacts and maintain a slice thickness ≤ 2 mm. We prepared an image series from four phases, namely, arterial, corticomedullary, nephrographic, and excretory, with 1-mm slice thickness and input them into the application (Figure 1).

### Step 2: Segmentation of the renal parenchyma

**Figure 2.**
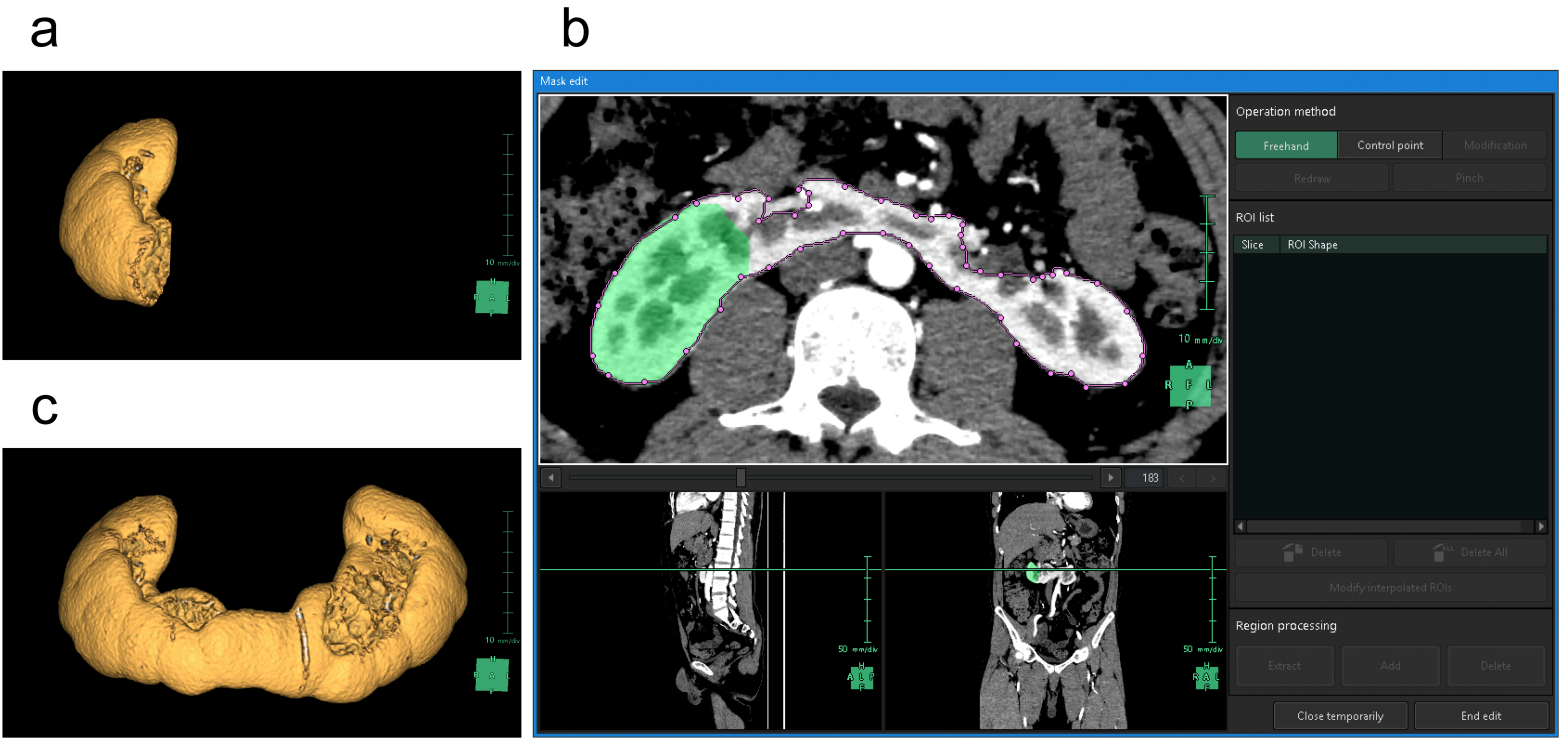
(a) Artificial intelligence (AI) reconstructs the 3D graphics of the right renal parenchyma. It is necessary to manually correct for the complete lack of recognition of the isthmus and inadvertent recognition of extraneous vessels at the hilum. (b) In the process of manual modification using the “Mask edit” tool, the regions of interest (ROI) within the renal parenchyma that the AI has chosen are filled in green. We can use a feature that closely resembles the Lasso tool of Adobe Photoshop and easily trace the ROIs not recognized by AI with purple dots and lines. (c) The 3D graphics reconstructed after manual modification.

For the segmentation of the renal parenchyma, either the arterial phase ^(5)^ or the corticomedullary phase is advantageous. Unless significant anomalies are present in the renal morphology, artificial intelligence (AI) applications can automatically segment the renal parenchyma in the CT images for each side separately and accurately reconstruct 3D graphics. However, if margins are unclear or have significant anomalies ^(9)^, the segmentation capabilities of AI may be limited, thus requiring manual adjustments ^(10)^. Using the “Mask edit” tool, we manually modified the margins of renal parenchyma, particularly the isthmus (the conjoint area between the left and right kidneys in horseshoe kidneys), which AI could not recognize (Figure 2). This tool enables manual tracing of the ROI in selected key sections of continuous CT and can subsequently connect the traced sections smoothly, thereby automatically filling in the ROI in the intervening sections. The tools provided by this application are similar to the “Lasso tool” of Adobe Photoshop, which enables segmentation of objects based on differences in image contrast ^(11)^.

### Step 3: Image registration

In this application, “Kidney Analysis” requires the completion of image registration after tracing of the renal parenchyma. Image registration involves the overlay of multiple images ^(12), (13)^. Even in a series of contrast-enhanced CT images of the same object, a proper image registration is necessary because of changes in positional relationships across different phases. During breathing, the kidneys significantly move in a craniocaudal direction within the trunk. To minimize this effect, we instructed the patients to maintain consistent inhalational volumes during CT imaging; however, such an approach has limitations ^(14)^. To adjust for the gap between relatively static structures, such as the skeleton, and more mobile structures, such as the kidneys, we employed nonrigid image registration. The “Kidney Analysis” application utilized in this study accurately aligned the structures from the hilum to the pelvis across the four phases of contrast-enhanced CT with high precision.

### Step 4: Segmentation of the arterial system

After image registration, the surrounding organs are extracted. Although SVSP supports vascular extraction from plain CT scans ^(15)^, it often encounters difficulties when applied to the kidneys. The arterial system is the most easily segmented as the earliest arterial phase enables selective high-contrast imaging of the arteries in enhanced CT. In “Kidney Analysis,” AI typically generates graphics of the abdominal aorta and renal arteries at the level of the kidneys. This limitation enabled us to avoid 3D reconstruction of the structures that were unnecessary for kidney observation, such as the celiac trunk, superior mesenteric artery (SMA), and inferior mesenteric artery.

**Figure 3.**
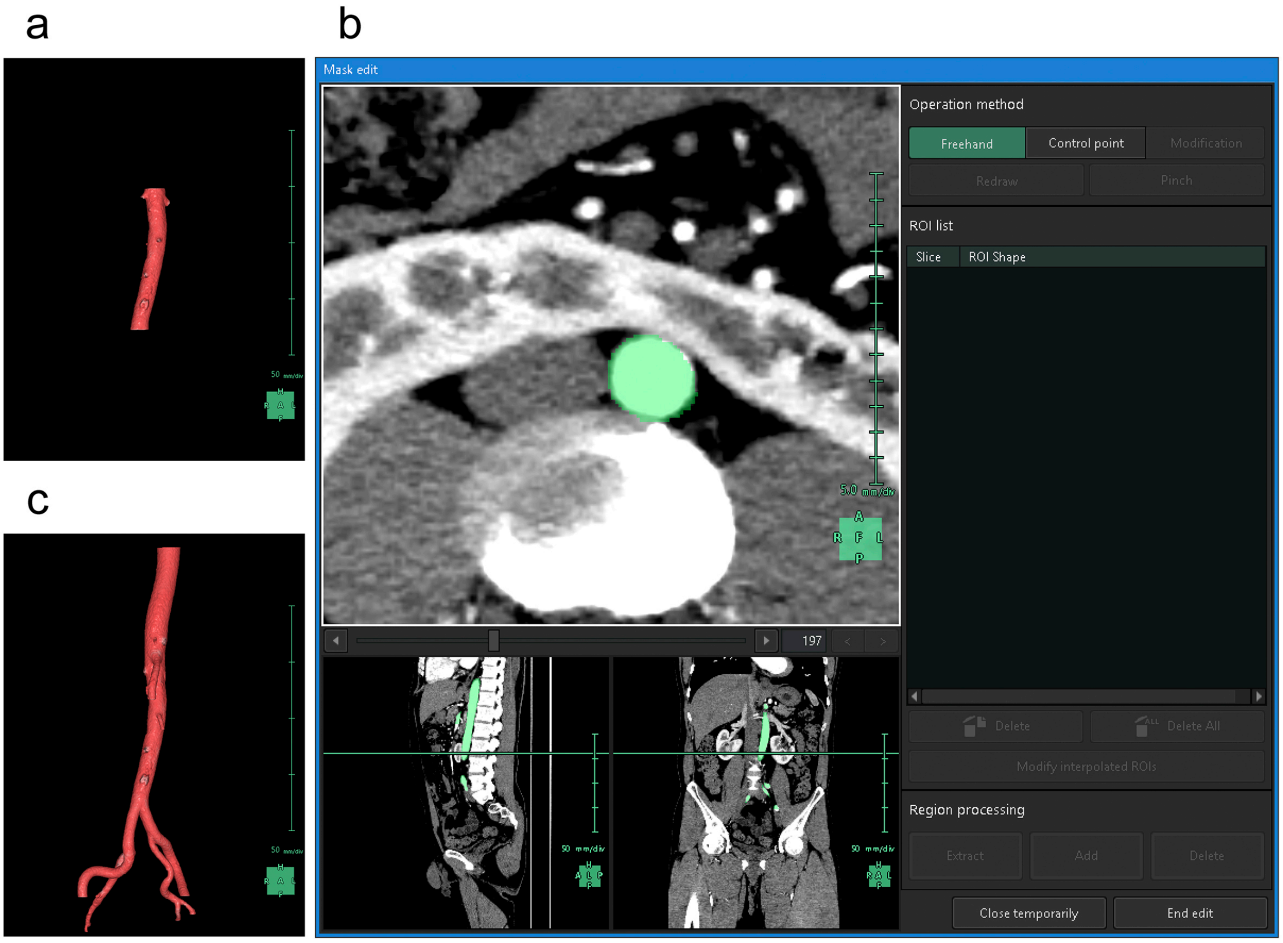
Reconstruction of the aorta. (a) Artificial intelligence (AI) reconstructs the 3D graphics of the abdominal aorta. The region of interest (ROI) is limited to the level of the kidneys, which results in graphics that omit the drawing of aortic branches. (b) Manual modification using the “Mask edit” tool. (c) 3D graphics reconstructed after manual modification. We added the common iliac artery, external iliac artery, internal iliac artery, and a portion of the superior mesenteric artery to the ROI.

To understand the spatial relationships of renal vessels with structures in the pelvis or external genitalia, we performed additional segmentation of the required structures using the “Mask edit” tool to segment relatively large arteries, such as the common, external, and internal iliac arteries, as structures that were continuous with the abdominal aorta. We occasionally segmented the SMA partially to examine and visualize the compression of the left renal vein between the abdominal aorta and the SMA (Figure 3).

**Figure 4.**
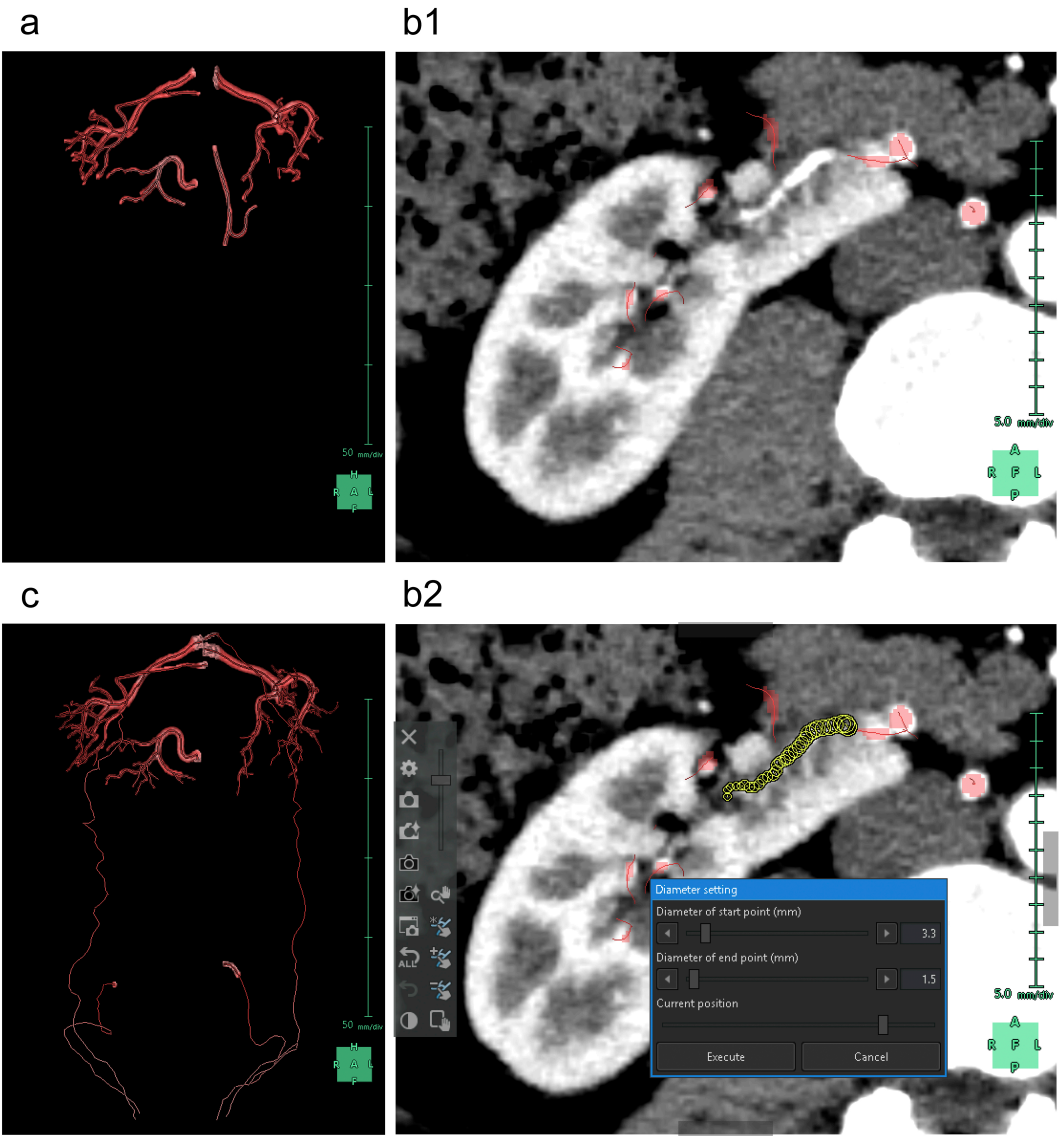
Reconstruction of the renal and testicular arteries. (a) Artificial intelligence (AI) reconstructs the 3D graphics of the renal arteries. (b) Manual modification using the “Diameter setting” tool (b1). When we specify the diameters and coordinates at the start and end points of an artery not segmented by the AI, a new artery segmented as a collection of yellow circles is added (b2). (c) 3D graphics reconstructed after manual modification. We removed the inferior mesenteric artery mistakenly segmented by the AI and newly segmented the fine network of blood vessels that supply blood to the testes.

Subsequently, we segmented the distal arteries. In “Kidney Analysis” applications, AI instantaneously and automatically segments renal arteries. However, its segmentation capability does not surpass that of experienced specialists ^(16), (17)^. Therefore, we performed additional manual segmentation. As the cross-section of elastic arteries resembles a circle, even arcuate and interlobar arteries, with their complex and varied paths, can be simplified into cylindrical models, essentially as paths of circles through space ^(18)^. The use of the “Diameter setting” tool of the application to define the diameters and coordinates at the start and end points enables precise 3D reconstruction that captures even the smallest peripheral branches ^(19)^. To comprehend the multiple arterial systems in the testicular blood supply, we utilized this tool to segment not only the renal arteries but also the fine arteries, which originate from the renal arteries, aorta, or internal/external iliac arteries (Figure 4).

**Figure 5.**
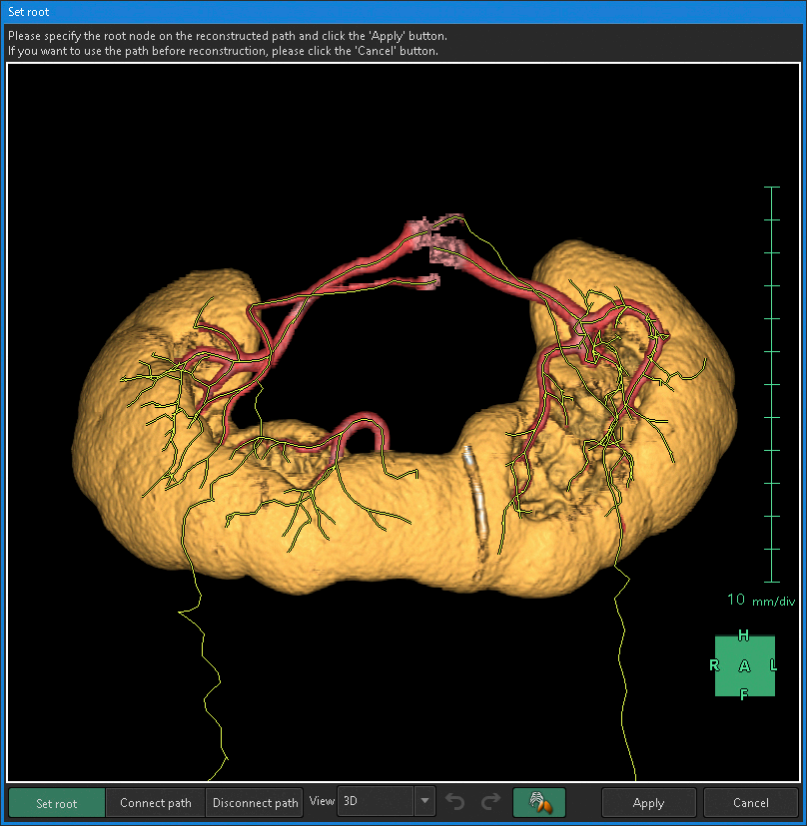
Specification of the root node on the reconstructed path. When the “Set Root” tool is activated, the root node of the arteries can be specified.

Any artery that carries blood, regardless of the size, should connect to the aorta when traced to its origin. Thus, a segmental artery that is not connected to the aorta indicates an error in the segmentation process. In this application, when setting a starting point for an artery, the AI automatically links it to nearby arteries according to the set threshold. Moreover, with the “Set Root” tool of the application, we can easily determine the main starting point of the peripheral arteries (Figure 5).

### Step 5: Segmentation of the venous system

Selective enhancement of only the venous system using CT imaging in either the corticomedullary or the nephrographic phase poses technical challenges. However, after complete selective segmentation of the arterial system, we can only segment the enhanced venous system as the AI of this application does not segment another object in the same location as the already segmented object. Therefore, we ensured that the segmentation of the arterial system was complete before initiating the segmentation of the venous system.

**Figure 6.**
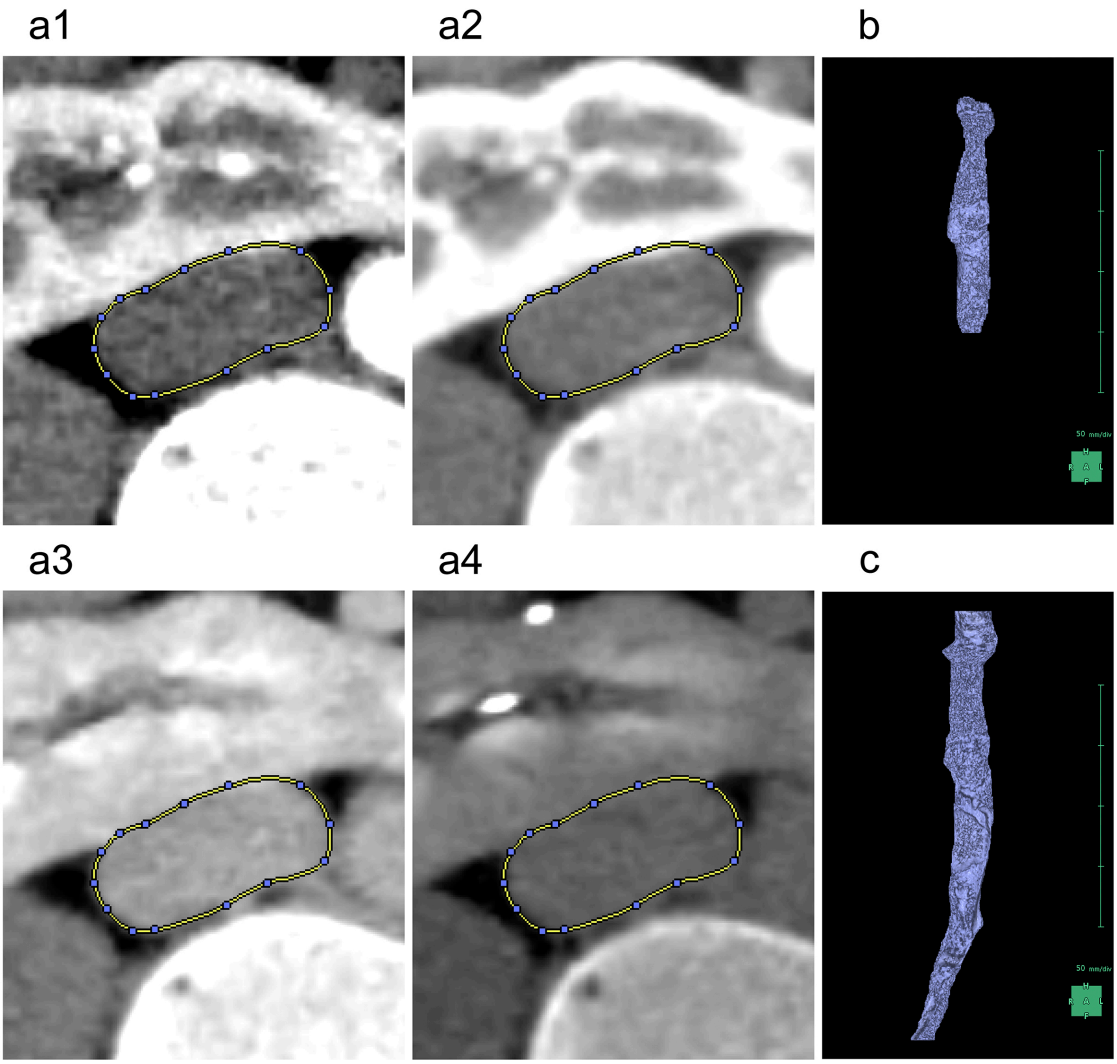
Reconstruction of the inferior vena cava (IVC). (a) Comparison of the contrast of the IVC at the level of the isthmus. In each of the four phases, namely, artery (a1), corticomedullary (a2), nephrographic (a3), and excretory (a4), we encircle the IVC as the region of interest (ROI) with blue dots and yellow lines. (b) Artificial intelligence (AI) reconstructs the 3D graphics of the IVC. The ROI is limited to the level of the kidneys. (c) 3D graphics reconstructed after manual modification. We added the right common iliac vein to the ROI.

When segmenting the inferior vena cava (IVC), the “Kidney Analysis” application features an AI that not only automatically segments but also provides a designated tool for manual segmentation. However, it is recommended to select between the corticomedullary and nephrographic phases based on contrast (Figure 6a) ^(5)^. This tool has functions that are almost equivalent to the previously mentioned “Mask edit” tool, although it differs in that it uses Bézier curves instead of the “Lasso tool.” Although the “Mask edit” tool enabled us to segment the aorta and accommodate branches beyond the left and right common iliac arteries, this IVC-designated tool did not support branching. Thus, we only segmented the right common iliac vein, which remained relatively straight from the IVC (Figure 6b and 6c).

**Figure 7.**
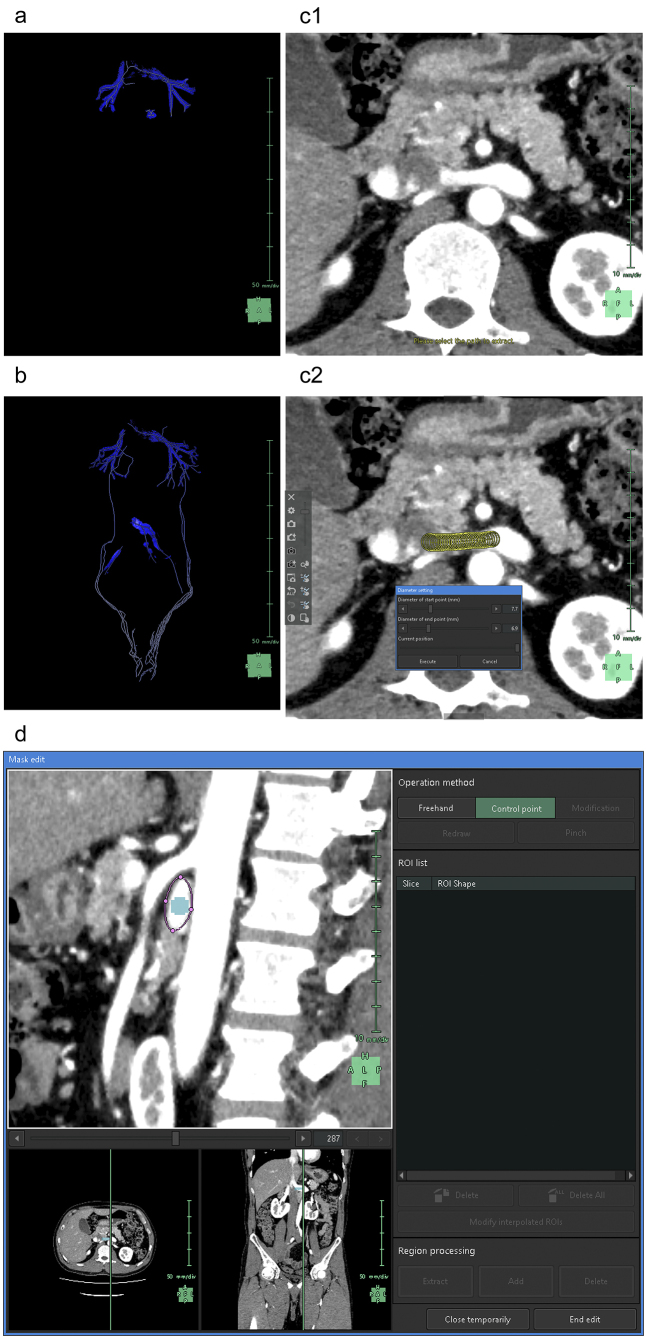
Reconstruction of the renal and testicular veins. (a) Artificial intelligence (AI) reconstructs the 3D graphics of the renal veins. (b) 3D graphics reconstructed after manual modification. We removed the artifact areas mistakenly segmented by the AI and newly segmented the pathway for blood return from the testes to the central circulation, including the left common iliac vein. (c) Segmentation process of the left renal vein compressed and deformed into a spindle shape between the aorta and the superior mesenteric artery (SMA). An axial image at the level where the left renal vein flows into the inferior vena cava (c1). We used the “Diameter setting” tool to set the diameters and coordinates at the start and end points of the left renal vein (c2). (d) Sagittal image at the level where the SMA branches from the aorta. As the “Diameter setting” tool alone cannot represent the irregular spindle-shaped cross-section of the left renal vein, we also used the “Mask edit” tool to add the region of interest of the left renal vein with purple dots and lines.

We then proceeded with the segmentation of the peripheral veins. In general, the AI in this application does not track the course of the veins as accurately as it does for arteries. Although initially segmenting the arteries may simplify the process to some extent as aforementioned, vein segmentation requires a more time-consuming and meticulous manual work ^(20)^. We segmented the renal veins, left common iliac vein, and veins returning blood from the testes to the central circulation (Figure 7a and 7b). Although the “Diameter setting” tool effectively segments the veins, the cross-sectional shape of the veins, which are elliptical or irregularly spindle-shaped, differs from that of the arteries. Thus, to achieve excellent 3D reconstruction, it is necessary to use the “Mask edit” tool to add vein contours to the ROI (Figure 7c and 7d).

### Step 6: Segmentation of the upper urinary tract

The urinary tract, which is possibly the easiest structure to segment following the arteries, offers distinct contrast in the excretory phase of contrast-enhanced CT and comprises a series of pathways, including the renal pelvis, ureter, bladder, and urethra. We herein describe our approach to segmenting the upper urinary tract, specifically the renal pelvis and ureter.

**Figure 8.**
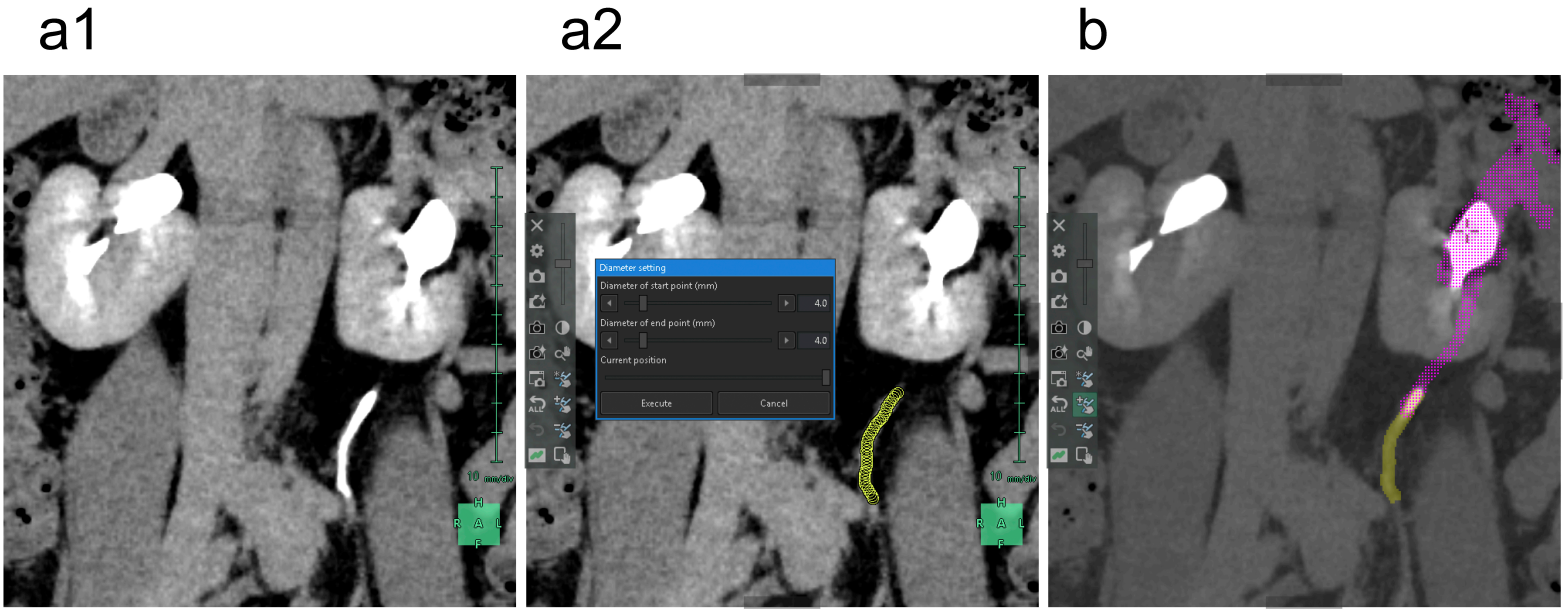
Reconstruction of the upper urinary tract. (a) Coronal section image where the course of the ureter is clearly visible during the excretory phase (a1). We used the “Diameter setting” tool to set the diameters and coordinates at the start and end points of the ureter (a2). (b) We adjusted the contrast to avoid mistakenly segmenting the solid structure of the renal pyramids, keeping the calyces and renal pelvis white while turning the renal pyramids black. When we used the “Region growing” tool starting from the center of the renal pelvis, the intricate structure of the renal pelvis, described as “coral” or “staghorn,” is depicted in purple.

First, the ureter, which has a tubular structure, can be simulated using a cylindrical model similar to that used for the arteries. Thus, we used the “Diameter setting” tool to segment the ureter. Although the AI in this application can automatically track and segment the ureter pathway ^(21)^, it tends to overestimate changes in diameter due to peristaltic movements. Consequently, we believe that manual segmentation by specialists who are familiar with this application often yields more natural 3D reconstructions (Figure 8a).

Subsequently, the renal pelvis was segmented. Urine in the kidneys flows from the apex of the renal pyramids through the renal papillae into cup-shaped minor calyces, which merge to form the major calyces that eventually lead to a funnel-shaped renal pelvis. Capturing of the appropriate excretory phase in an enhanced CT scan enables the observation of a relatively strong and heterogeneous contrast effect in the renal pyramids and a significantly stronger and homogeneous contrast effect from the calyces to the renal pelvis. To differentiate between the solid structure of the renal pyramids and urine-filled void spaces of the calyces and renal pelvis, we adjusted the image contrast and brightness. This enabled us to darken the renal pyramids and brighten the calyces and renal pelvis by applying the “Region growing” tool to this adjusted image for segmentation of areas with the same contrast in the calyces and renal pelvis (Figure 8b) ^(22)^. This method is rapid and accurate in patients without hydronephrosis. In cases of hydronephrosis, homogeneously filling an enlarged renal pelvis with a urine-containing contrast agent is challenging. Thus, to segment unenhanced areas of the renal pelvis, we used not only the “Region growing” tool but also the “Mask edit” tool.

Notably, segmentation of the ureter after the renal pelvis can cause the AI to invalidate the segmented areas generated using the “Region growing” or “Mask edit” tool. Thus, we completed the segmentation of the entire ureter before moving on to the renal pelvis. Similarly, when using the “Set root” tool, the AI can invalidate the segmented areas of the renal pelvis. Therefore, we avoided using the “Set root” tool for the ureter and renal pelvis.

### Step 7: Settings for the rendering of the arteries and veins

**Figure 9.**
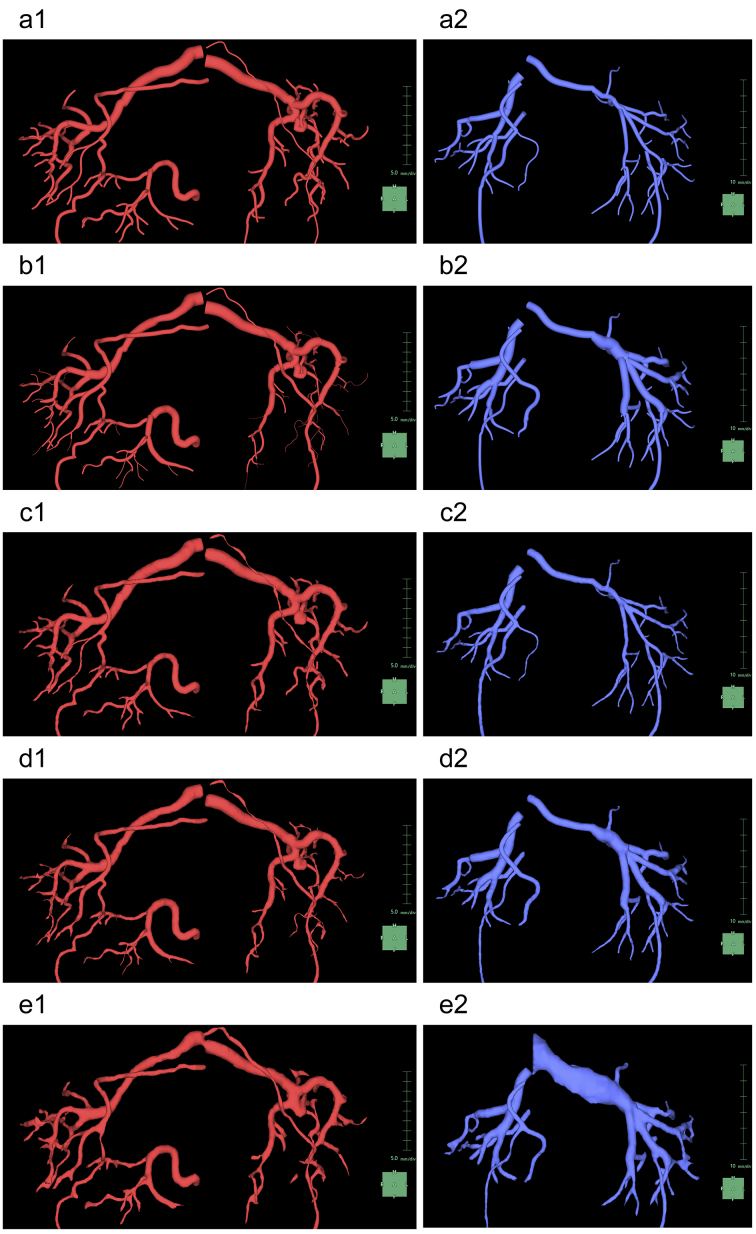
Display of the arteries and vessels for each rendering setting provided by the application. Arteries are depicted in red (a-e1) and veins in blue (a-e2). (a) “Cylinders” mode and “Monotonically decreased” mode. (b) “Cylinders” mode and “Estimated diameter from image” mode. (c) “Region” mode and “Monotonically decreased” mode. (d) “Region” mode and “Estimated diameter from image” mode. (e) “Keep image” mode. Note that in this mode, there is no difference in display between “Cylinders” mode and “Region” mode. Furthermore, it is the only mode where the segmentation complemented by the “Mask Edit” tool is displayed.

When displayed in 3D, there is an option to choose between volume and surface rendering, with the latter selected owing to its ability to present structures more smoothly than volume rendering ^(23)^. In this application, surface rendering facilitates easier understanding of the spatial relationships of vessels ^(19)^, making it the preferred choice. When surface rendering vessels, this application offers separate settings for “Shape of vessel surface” and “Type of vessel surface” for the arteries and veins, respectively (Figure 9). In general, for “Shape of vessel surface,” the “Region” mode provides a more natural display than “Cylinders” mode. For “Type of vessel surface,” we select from “Monotonically decreased,” “Estimated diameter from image,” and “Keep image” modes. Although we often select “Estimated diameter from the image” mode for arteries owing to its aesthetic display, the difference is minimal as long as careful segmentation is performed using the “Diameter setting” tool. However, for veins, we must choose the “Keep image” mode as it is the only option that enables the AI to display segments complemented by the “Mask edit” tool segmentation for veins with noncircular cross-sections.

### Step 8: Observing the final product

**Figure 10.**
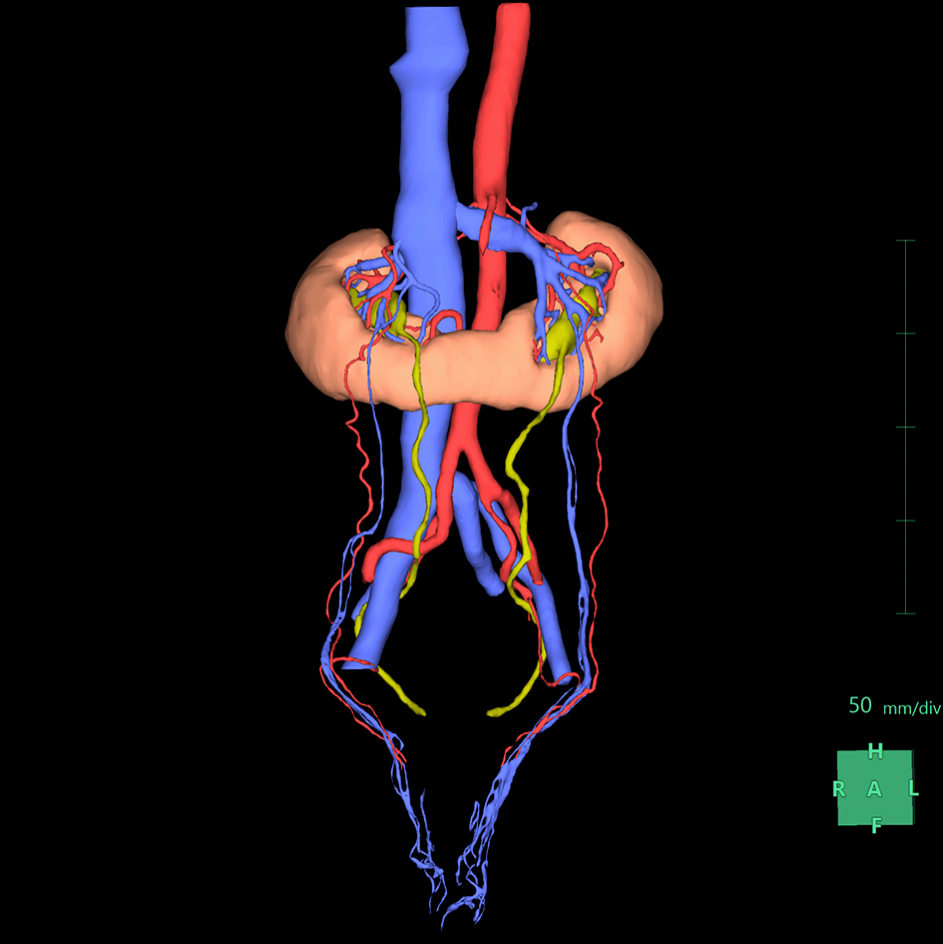
Final display provided by the application. The application automatically color-codes the display: renal parenchyma in light orange, arterial system in red, venous system in blue, and upper urinary tract in yellow. In addition, the application clearly depicts not only the area near the kidneys but also the varicoceles.

Finally, we proceed to “Observation.” This application provides a color-coded display of the areas that we sequentially segmented in the previous steps, supporting our deep and comprehensive understanding of anatomical structures (Figure 10). In addition, the SVSP functions to export a 3D PDF that is accessible even on computer terminals where the SVSP is not installed ^(24)^, significantly facilitating patients in visualizing and understanding their condition and enabling them to focus on the ongoing infertility treatments. The entire process, from the initial steps to the export of the 3D PDF, takes approximately 2 h. The 3D PDF is available as a supplementary file, making it suitable for attachment and publication in manuscripts like this one.

3D PDFs can be opened with free software such as Adobe Acrobat Reader. Note that 3D PDFs cannot be viewed with standard web browsers like Edge, Google Chrome, or Firefox. Viewing requires software capable of displaying 3D content, such as Adobe Acrobat Reader. Ensure the latest version is installed. Upon opening the file, activate the 3D content by clicking the designated button.

3D PDFs adhere to ISO 32000-2 standards, ensuring compatibility and interoperability across different systems and software. Users can interact with 3D models by dragging to rotate and view from different angles. This interaction allows detailed examination of the model, simulating the effect of physically manipulating the object. Users can zoom in and out, pan across the model, and spin it on various axes.

For detailed instructions on displaying 3D models in PDFs, refer to the Adobe support page (last updated on Oct 31, 2023, retrieved on May 20, 2024): https://helpx.adobe.com/acrobat/using/displaying-3d-models-pdfs.html. More information on PDF standards can be found on the PDF Association website (last updated on May 16, 2024, retrieved on May 20, 2024): https://pdfa.org/sponsored-standards/.

## Discussion

We herein describe the process of 3D rendering of horseshoe kidneys accompanied by bilateral varicoceles using the “Kidney Analysis” application, a feature of SVSP. Although this application offers highly effective automatic segmentation functionalities specifically designated for kidneys, it does not support anomalies with severe irregularities, such as horseshoe kidneys. Nevertheless, by leveraging the “Mask edit,” “Diameter setting,” and “Region growing” tools, we successfully overcame this limitation, obtaining a display that was both aesthetically pleasing and anatomically accurate. Thus, by achieving proficiency in using only these three tools, we can visualize any anatomical abnormality without the need for specialized knowledge of 3D graphics.

Horseshoe kidney is a relatively rare anomaly found in approximately 0.25% of the population ^(25)^. Varicocele, a condition that is characterized by the presence of dilated veins within the spermatic cord, affects approximately 15% of adult men and is one of the most prominent causes of male infertility ^(26)^. Although varicoceles are typically painless, rare cases similar to the present case, in which patients experience pain during ejaculation, have been reported ^(27)^. Moreover, as in this present case, the presence of a varicocele on the right side requires screening for retroperitoneal tumors, including renal cancer ^(28)^. Although both horseshoe kidneys and varicoceles are well-known abnormalities in urology, there is currently no available literature on their coexistence. Furthermore, Kartagener syndrome, which was ruled out in the present case, was a significant concern during the progression of infertility treatment for the patient. However, there is no available literature that adequately explains any pathophysiological relationship between horseshoe kidney and Kartagener syndrome. Previous reviews on horseshoe kidneys have reported complications, such as urinary tract obstruction ^(29)^, absence of vas deferens ^(30)^, and severe vascular anomalies ^(31)^. In particular, varicocele associated with the Nutcracker phenomenon, frequently observed in conjunction with horseshoe kidney ^(32)^, is a plausible pathology. However, its frequency and actual prevalence remain unknown. Varicoceles are strongly associated with increased pressure in the renal vein ^(33)^. Thus, by accumulating 3D displays of renal veins in cases such as horseshoe kidneys, insights into the relationship between horseshoe kidneys and varicocele may be obtained.

Medical illustration is advantageous for various purposes, including preoperative evaluations, intraoperative records, postreflection, medical education, noninvasive systematic anatomy, and clinical considerations of typical or atypical cases ^(34)^. The effectiveness of 3D rendering as an anatomical reference was first reported in the 20^th^ century, particularly with advancements in computer technology ^(35)^. In recent years, 3D rendering has not only been advantageous among experts but has also facilitated communication between patients and healthcare providers ^(2), (36)^. This report demonstrates clear 3D renderings of the underlying condition of a male patient with infertility. Such renderings helped alleviate the patient’s unnecessary worries and concerns, allowing him to focus on his infertility treatment. The importance of voluntary involvement in medical illustration by actively engaging in hands-on work was mentioned in as early as the 1960s ^(37)^. However, because of increasing healthcare specialization, medical illustrations are considered to be the domain of trained professionals ^(38)^.

By adopting popular consumer software that is often used for daily activities like presentations and image editing within business, government, healthcare, and educational settings, tracing medical images can be viewed as a practical solution that is readily accessible to medical professionals lacking artistic skills. Attempts at tracing using tools such as Microsoft PowerPoint ^(39)^ and Adobe Photoshop ^(11)^ have been reported. The semiautomatic segmentation feature of SVSP is appealing owing to its intuitive operation, akin to tracing organs with tracing paper, enabling the generation of exquisite images within approximately 15 ^(21)^ to 60 ^(20)^ min with proficiency.

The history of SVSP has been mainly centered on preoperative evaluations in gastrointestinal surgery ^(20)^, with the subsequent recognition of its utility in thoracic surgery. Following the first documented use of SVSP in liver surgery published in 2012 ^(40)^, SVSP has been used for procedures involving the liver ^(3), (41), (42), (43)^, pancreas ^(17), (44)^, colon ^(45)^, rectum ^(46), (47)^, stomach ^(48), (49)^, bronchus ^(16), (50), (51), (52)^, aorta ^(53), (54)^, diaphragm ^(55)^, and thorax ^(56), (57)^. While there have been reports of its use in urological surgeries, such as partial nephrectomy ^(58), (59), (60), (61), (62), (63)^, ectopic varices in ileal conduit ^(64)^, and retroperitoneal lymph node dissection for testicular tumors ^(65)^, such applications have mostly been limited to the quantification of the volume of the renal parenchyma ^(66), (67), (68), (69), (70), (71), (72)^, perirenal fat ^(73), (74)^, psoas muscle ^(75)^, urination-related visceral fat ^(76)^, and prostate ^(77)^. Consequently, urologists have underutilized the strengths of SVSP in displaying intricate 3D vascular anatomy.

According to the results of an international survey reported in 2023, more than half of all urologists acknowledge the utility of 3D rendering. However, because of cost and time constraints, less than 25% of them actually applied it in clinical practice ^(78)^. As of 2018, over 1,000 facilities in Japan have implemented SVSP ^(79)^. We hope that upon reviewing this report, urologists will gain broader recognition of the effectiveness of SVSP. This software, obtainable in approximately 2 weeks ^(6)^, offers significant benefits through its simple operation. However, to achieve this, it is important to address the challenges associated with SVSP. First, the implementation of the application is expensive ^(20)^. Second, typical challenges include interuser inconsistencies due to variations in proficiency, changes in contrast, and differences in imaging conditions, which can decrease the effectiveness of automatic segmentation functions ^(43)^. Furthermore, we emphasize that manual intervention is indispensable for vein segmentation ^(20)^. Although one report suggested that better results can be obtained when anatomy experts spend a full day meticulously outlining structures without relying on AI during segmentation ^(52)^, it is also pivotal to determine the value of investing such efforts during busy clinical duties.

In conclusion, the integration of 3D rendering techniques via the “Kidney Analysis” application in SVSP has shown promise in visualizing complex anatomical anomalies, such as horseshoe kidneys with bilateral varicoceles. Despite the challenges, we overcame the limitations of SVSP tools and achieved accurate and visually appealing displays. Our findings highlight the proficient use of SVSP tools for visualizing diverse anatomical abnormalities without knowledge on specialized 3D graphics.

## Article Information

### Conflicts of Interest

None

### Author Contributions

All authors fulfill the ICMJE authorship criteria

### Approval by Institutional Review Board (IRB)

Approval number: R03-033, Institutional Review Board of the University Tsukuba Hospital

### Informed Consent

Informed consent was obtained from the patient.

## Supplement

Supplementary Material
