# Supplementary material for: Practical Step-by-step SYNAPSE VINCENT Rendering of Three-dimensional Graphics in Horseshoe Kidney with Bilateral Varicoceles
Source: JMA J. 2024 Aug 9;7(4):471–86. doi: 10.31662/jmaj.2024-0058 (PMC11543326; doi:10.31662/jmaj.2024-0058)
Supplement: Supplementary Material [file 2433-3298-7-4-0471-s001.pdf]

Horseshoe kidney with bilateral varicoceles

Region	Volume	View
Total kidney	361.4 ml	
Total cortex	292.4 ml (80.9 %)	
Horseshoe kidney	361.4 ml (100.0 %)	
Artery	—-	
Vein	—-	
Upper urinary tract	—-	
Inferior vena cava	—-	
Aorta	—-	
